# A Clinical Primer on Computer Vision

**DOI:** 10.1007/s00223-026-01620-9

**Published:** 2026-10-07

**Authors:** Jon Parkinson, Claudia Lindner

**Affiliations:** Division of Informatics, Imaging and Data Sciences, The University of Manchester, Manchester, UK

**Keywords:** Computer vision, Medical image analysis, Machine learning, Artificial intelligence, Clinical translation

## Abstract

Computer vision is a rapidly evolving field within computer science that focuses on extracting meaningful information from digital images. In medical imaging, computer vision techniques such as object detection, classification, and segmentation are essential for analysing complex anatomical structures. This primer provides an overview of computer vision concepts and methods, highlighting both traditional approaches and modern machine learning techniques. Landmark detection and shape modelling are also discussed as these are particularly relevant for musculoskeletal imaging. Challenges associated with applying computer vision in clinical practice are examined. This includes considerations such as data limitations, domain shift, and the need for robust validation. Finally, future directions for integrating computer vision into osteoporosis research and clinical workflows are explored, emphasising the importance of explainability, fairness, and adherence to guiding principles for trustworthy clinical tools.

## Introduction to Computer Vision

Computer vision is the field of computer science concerned with the extraction and recognition of meaningful information from digital images. Common computer vision tasks in medical imaging include detecting the location and shape of objects of interest, classifying objects present in an image, and segmenting medical images into precisely defined regions [1]. Computer vision tasks are applied across a range of medical imaging modalities, most commonly with a focus on Computed Tomography (CT), Magnetic Resonance Imaging (MRI) or X-ray.

Although computer vision has existed since the 1960s, it has surged in importance in recent years due to various factors, including the availability of huge quantities of data, advances in computational processing power, and significant algorithmic improvements. However, the deployment and adoption of computer vision within the healthcare sector is affected by a range of unique sociological and technical factors such as regulatory requirements, varying levels of clinical acceptance, and the relative maturity of the tools. Under the broader umbrella of Artificial Intelligence (AI), computer vision in radiology has become one of the most widely adopted technologies [2].

Potential applications of computer vision in the healthcare sector are extensive and include automatic trauma and pathology detection in radiology, for example, automating the diagnosis of fragility fractures and osteoporosis [3, 4]. In clinical practice, computer vision systems in radiology are typically deployed as decision-support tools, complimenting rather than replacing human expertise [5].

## Foundational Concepts

**Fig. 1:**
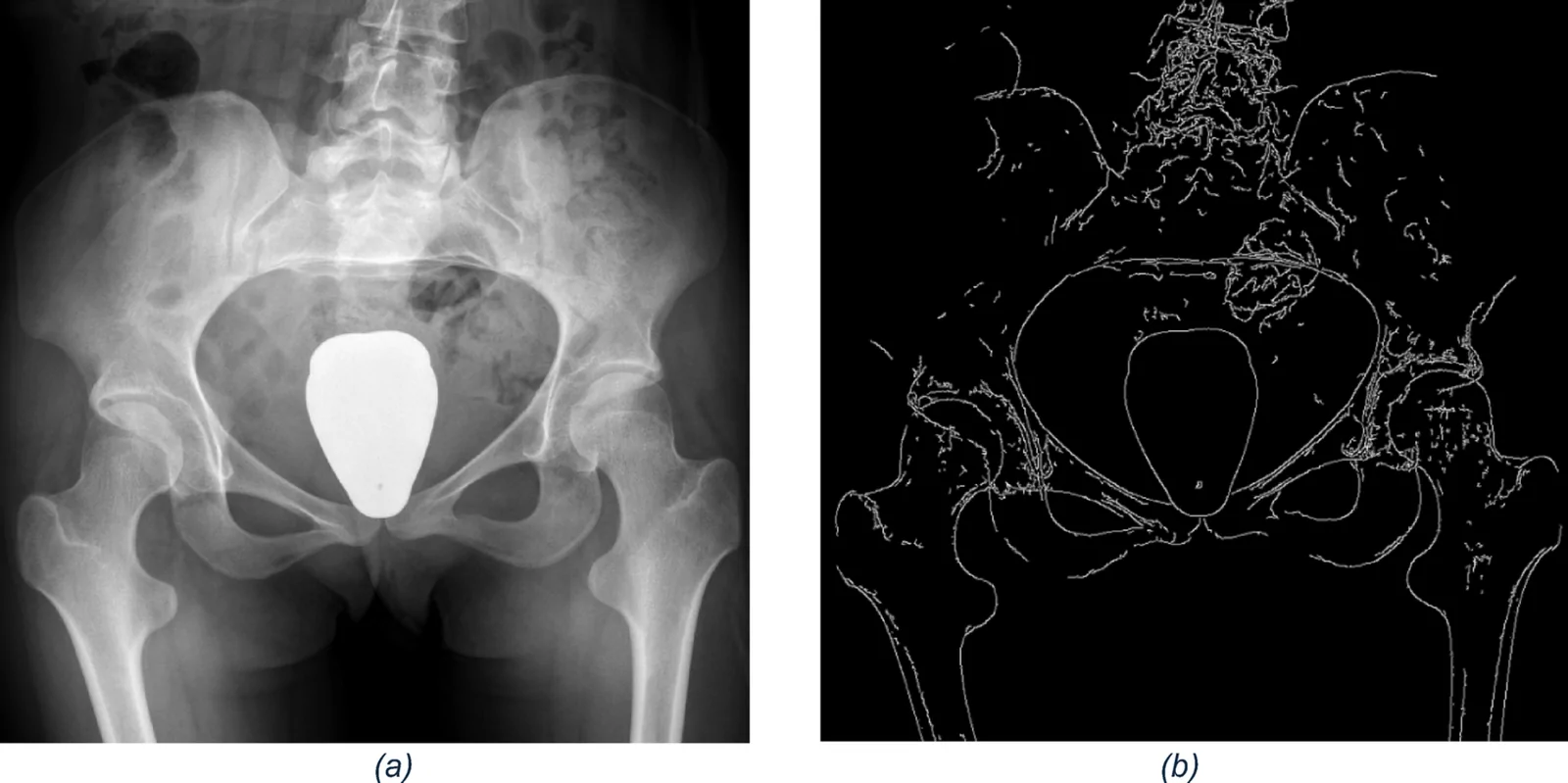
Example of edge detection: (***a***) Pelvic radiograph. (***b***) Output from running the Canny edge detection algorithm over the pelvic radiograph

Computer vision functions by identifying commonly occurring patterns of interest within the pixels comprising digital images. A simple example is the search for abrupt changes in the brightness of pixels (also known as pixel intensity) indicating the edges of objects. Finding edges in images can be manipulated to identify the shape of structures of interest. For instance, the contours of the hips shown in Fig. 1a are obvious to the human eye due to the abrupt change in pixel intensity at the edges of the skeletal structure. Humans detect these edges without effort. Computer vision provides methods to automatically detect these edges in digital images. One approach to this task is to scan an image looking for patterns indicating the presence of edges. This process is called edge-detection and enables the identification of structural limits of objects. The patterns of interest are known as *features* in the computer vision vernacular. Features may include (i) edges, where there is obvious change in pixel intensity or colour; (ii) combinations of edges forming corners; and (iii) ‘blobs’, which are regions with similar intensity or texture (i.e. the spatial arrangement of intensity values).

There are a wide variety of computer vision methods, and these are generally considered to fall within two broad categories, traditional computer vision methods, where feature detectors are hand-designed using prior human knowledge, and modern machine learning (ML) methods, where features are discovered automatically from data. A classic example of a traditional computer vision method is the Canny edge detector [6]. This algorithm functions via multi-stage processing of an image. After the image is processed to remove noise, edge-detecting feature detectors seek edges present in the image, followed by the removal of weak edges (see Fig. 1b). The feature detectors are applied as mathematical functions, interrogating small patches of pixels across the image to discover patches exhibiting the sought after characteristics. In the case of edge detection, these may take the form of a significant change in pixel intensity between the left and right, or top and bottom, of a patch, indicating the presence of an edge.

**Fig. 2:**
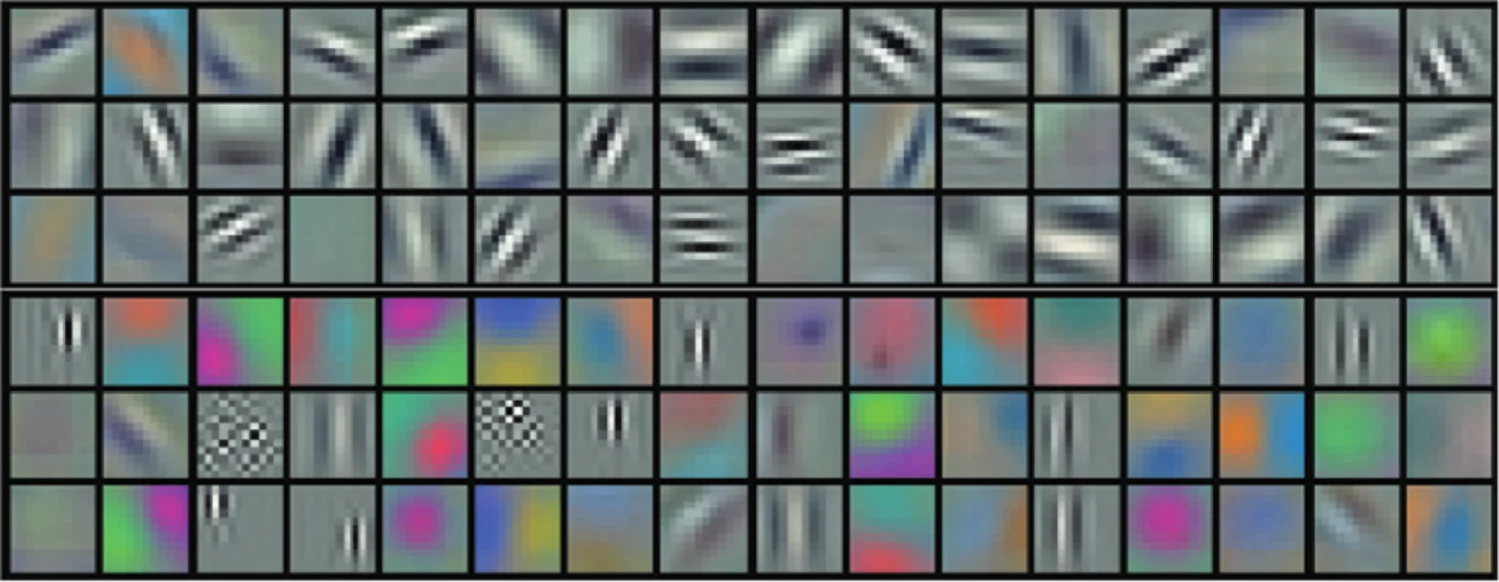
A summary of the features learned automatically from the ImageNet dataset by the Convolutional Neural Network commonly known as AlexNet; reprinted from [7; Fig. 3], with permission from the authors [7]

Modern ML methods use a different approach to feature design, learning their feature sets (kernels) automatically by interrogating large quantities of image data to discover regularly occurring patterns. A famous example of this is the set of 96 features learned by AlexNet, shown in Fig. 2 [7]. The range of features, including edges at varying angles and blob textures, were learned by automatically scanning the dataset and capturing regularly occurring patterns in small regions of 11 × 11 pixels sampled from ImageNet, a very large dataset containing over 14 million high-resolution real-world photographs [8].

One of the most useful aspects of modern ML computer vision is the option to adapt certain already-built ML models (i.e. already developed using very large quantities of image data) to perform specific user-defined custom tasks. To build a ML model completely from scratch can take significant time and resource, so it is often more efficient to update an existing model with data specific to the custom user task [9, 10]. This process, known as *Transfer Learning*, takes an existing model such as AlexNet for object detection, or U-Net for segmentation problems [11], and makes minor adaptions to the existing knowledge encoded in the original model so it performs well on a new task, saving time and cost [12].

As modern ML models tend to supersede the performance of traditional methods, in the following the focus will be predominantly on the former. However, there remains scope for traditional methods where existing human knowledge can be explicitly encoded into the feature selection process [13]. This is particularly the case in applications where datasets are small or where explainability and transparent decision-making are required.

## Core Computer Vision Tasks (Both Traditional and Machine Learning)

This section will cover three of the main computer vision tasks in turn, object detection, classification, and segmentation.

### Object Detection

**Fig. 3:**
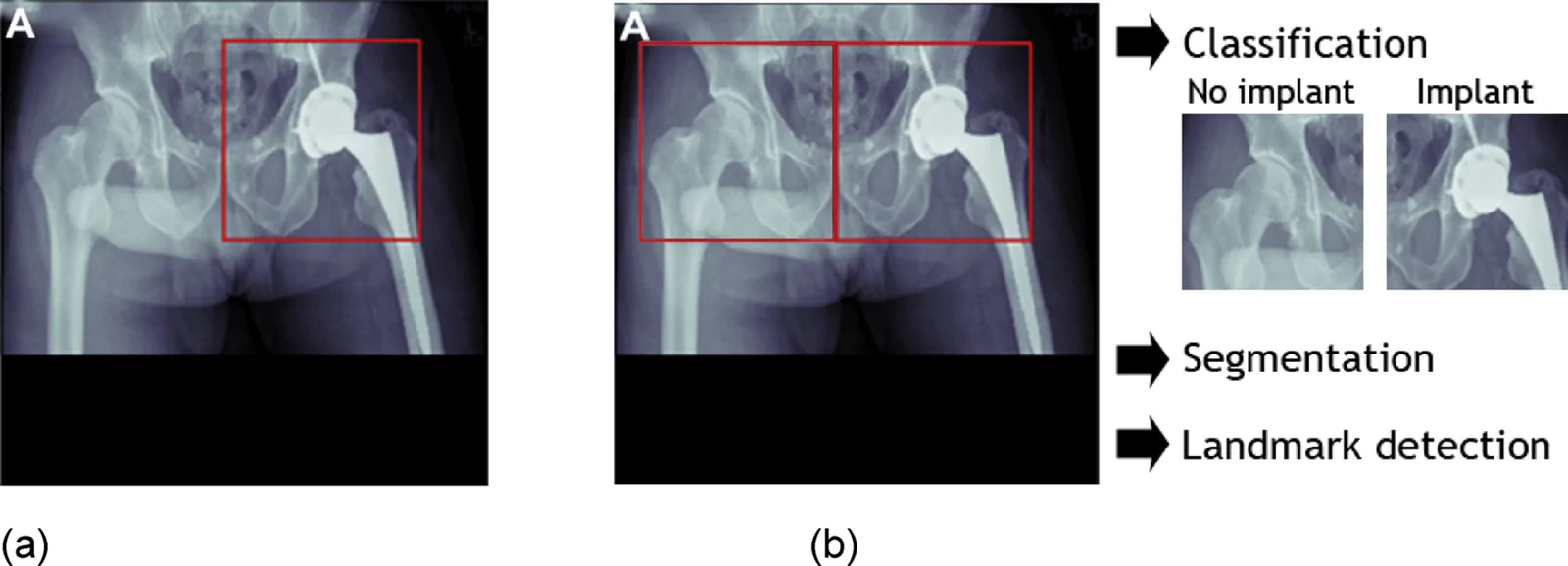
Object detection example: **a** Bounding box localising a hip joint in pelvic radiograph, detected with a YOLO-V3 model; reprinted from [18; Fig. 2A], with permission from Elsevier. **b** Illustration of multi-stage process with object detection providing the input for the application of further computer vision methods

The task of discovering objects of interest present in an image and learning the semantic labels associated with their class type is known as object detection. The output of an object detection task is often similar to that shown in Fig. 3a, where objects of interest are identified within their own bounding box (i.e. a rectangular box placed around an object of interest with all parts of the object existing within its corners). There are various methods to accomplish this task, but they all involve searching the image for features indicating the presence of the object of interest, such as the general shape of a hip joint implant viewed from different angles. The output of an object detection task is often used for further computer vision tasks as illustrated in Fig. 3b.

Traditional methods for object detection include Haar Cascades [14], which use hand-crafted Haar-like features and a sliding window approach (i.e. systematically moving a fixed-sized box across an image, scanning and analysing the sub-image at each box position for target objects) to evaluate small patches of an image individually for the presence of the object of interest. However, these tasks are now generally carried out by more powerful modern ML-based methods such as the R-CNN (Region-based Convolutional Neural Networks) [15], the YOLO (You Only Look Once) algorithm [16] or, more recently, Transformer-based vision algorithms, which are context-aware attention-based algorithms applicable to a range of tasks and which were first introduced with the ViT (Vision Transformer) model [17].

Object detection involves multiple challenges. The number of the objects of interest is often unknown in advance, and the detection of objects can be difficult in low resolution images or when objects of interest are occluded. For example, in pelvic radiographs, it may not be known in advance whether both hips contain implants or whether some anatomical structures are obscured by a gonad shield. Solving this challenge is of particular importance in the case of vertebral fracture detection as the quantity and location of the vertebrae in a candidate image is often not known prior to image processing. Each of the individual vertebrae must first be located before a diagnosis based upon analysis of their individual shape can be made [19].

### Classification

Classification is the process of determining the nature of something. When the goal is to assign a single label describing the content of an entire image, this is referred to as image classification; whereas when the goal is to identify the nature of individual objects within the image, this is known as object classification. If we consider Fig. 3a, rather than learning the location of a hip joint, the task may be to classify whether the patient has had hip replacement surgery. Using a traditional computer vision approach, this may be achieved by comparing image regions with a predefined template, a process known as template matching. Furthermore, object classification can involve determining a general label for objects detected, or assigning a category associated with each object. For example, Fig. 3b illustrates the classification of hips with versus without implants. The task at hand will determine which approach is appropriate.

Popular ML-based algorithms for image classification include the AlexNet introduced, and the YOLO algorithm introduced above, and the Deep Residual Learning Framework, commonly known as ResNet, which is a Convolutional Neural Network that can be fine-tuned for specific user tasks [ in Sect. 2^20^].

Classification in medical imaging has several potential obstacles that must be considered. A major challenge is the labelling of training data. For example, human experts may fail to agree on the diagnosis present in an image, making it difficult to establish a reliable ground truth. Another common issue is domain shift: classification models trained on data from specific scanners often exhibit degraded performance when applied to images from different scanners, especially when the scanner manufacturer and model is different to that used for creating the training data [21].

The diagnosis of osteoporosis from a detected object (e.g. spine) is considered a classification task, often using bone mineral density (BMD) as an indicator of the stage of bone loss, categorising it as osteopenia, osteoporosis or severe osteoporosis [22].

### Segmentation

**Fig. 4:**
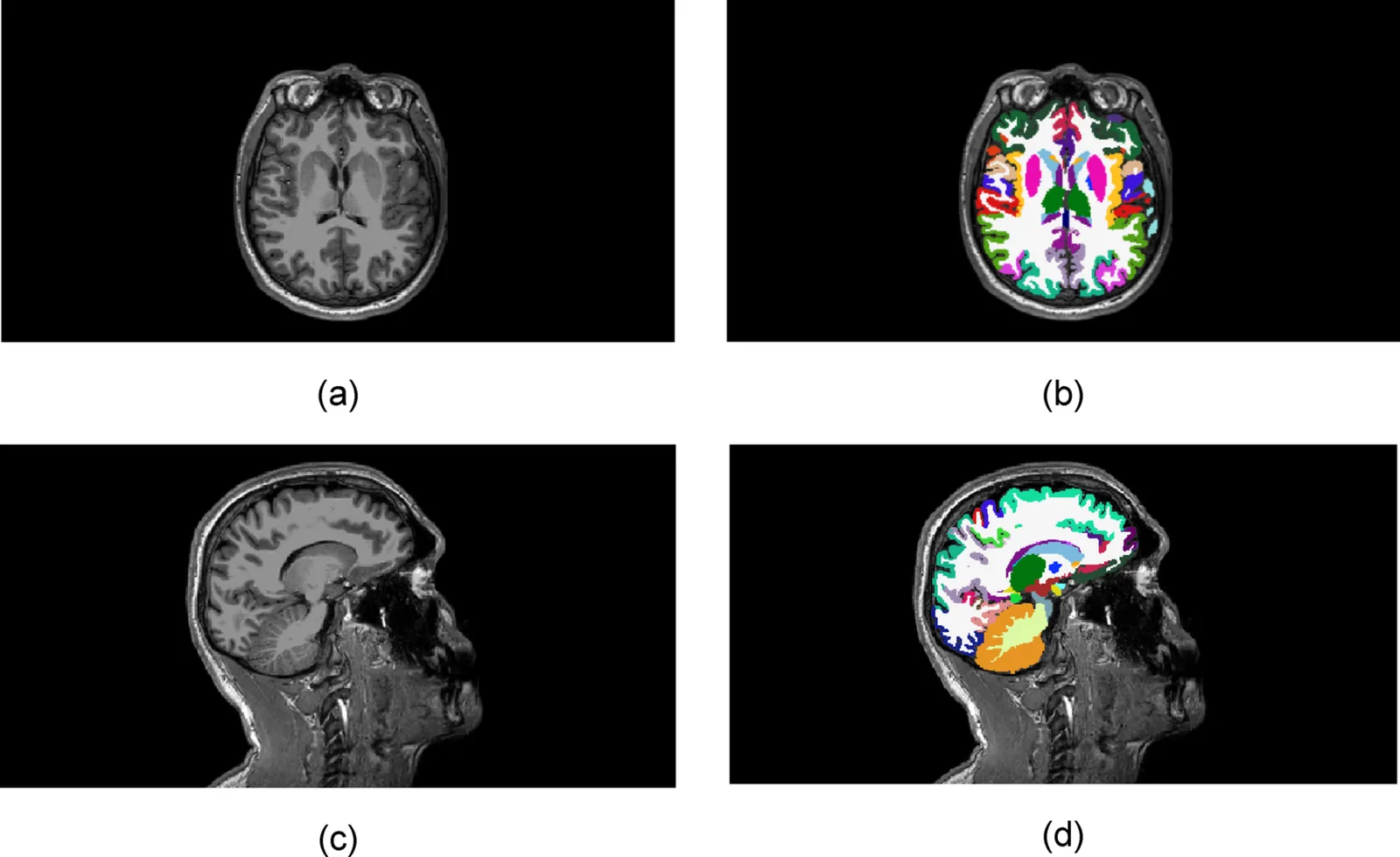
Segmentation example: (**a**) & (**c**) Axial and coronal plane images of a human brain taken from high-resolution Magnetic Resonance (MR) data (T1-weighted). (**b**) & (**d**) the same images segmented using the FreeSurfer software package to determine the different regions of the brain [24]

Where detection and classification seek to locate and/or identify objects, segmentation refers to the process of partitioning an image into regions of interest (ROIs) that exhibit similar characteristics. These could be regions of pixels belonging to the same object, such as the hip or spine, or to broader anatomical regions, such as those associated with organs, tissue, or skeletal structures. Segmentation can be used to partition the whole image or just a subset. The latter is illustrated in Fig. 4, which shows the output of segmenting the brain into distinct regions. Once this information has been extracted based on properties including the similarity in colour, the lightness or darkness of individual pixels (i.e. pixel intensity), or the texture of the ROIs, it can be used in subsequent downstream tasks such as disease diagnosis, volumetric measurement of organs, surgical planning, and more [23].

There is a range of segmentation strategies to choose from: (i) threshold-based segmentation assigns individual pixels to categories based on their relationships to one or more pixel intensity thresholds [1]; (ii) edge-based segmentation identifies the outer boundaries of regions using edge-detection methods similar to those introduced above [25]; and (iii) region-growing segmentation uses a set of seed points (i.e. initial candidate locations in an image to start searching, selected either manually or automatically) to mark distinct ROIs, which are subsequently expanded according to which pixels are likely to belong to the same region [26].

Two commonly applied ML-based segmentation algorithms in medical imaging are U-Net and MedSAM. U-Net is a neural network specifically designed for medical image segmentation, which can be downloaded and fine-tuned to problem-specific needs [11]. The more recent MedSAM (Medical Segment Anything Model) is a ML model designed to work across multiple imaging tasks, from medical images to images of everyday objects, and it uses an initial user prompt to discover the ROIs [27].

Segmentation in medical imaging comes with a set of unique challenges. The outer boundaries of anatomical structures often appear weak, especially in lower-contrast imaging modalities (e.g., X-ray compared to MRI). Moreover, the precise location of object boundaries in an image can be subject to disagreement among clinical experts, rendering it difficult to create accurate datasets for both developing and testing segmentation algorithms.

## Landmark Detection and Shape Modelling

Statistical Shape Models (SSM) [28] capture the range of shape variation within specific classes of objects. For example, while individuals may have slightly different shaped knee bones, there is a constrained range of anatomical variation. By building an SSM capturing the range of shape of a healthy bone, deviations away from this norm can be used to indicate potential pathology.

**Fig. 5:**
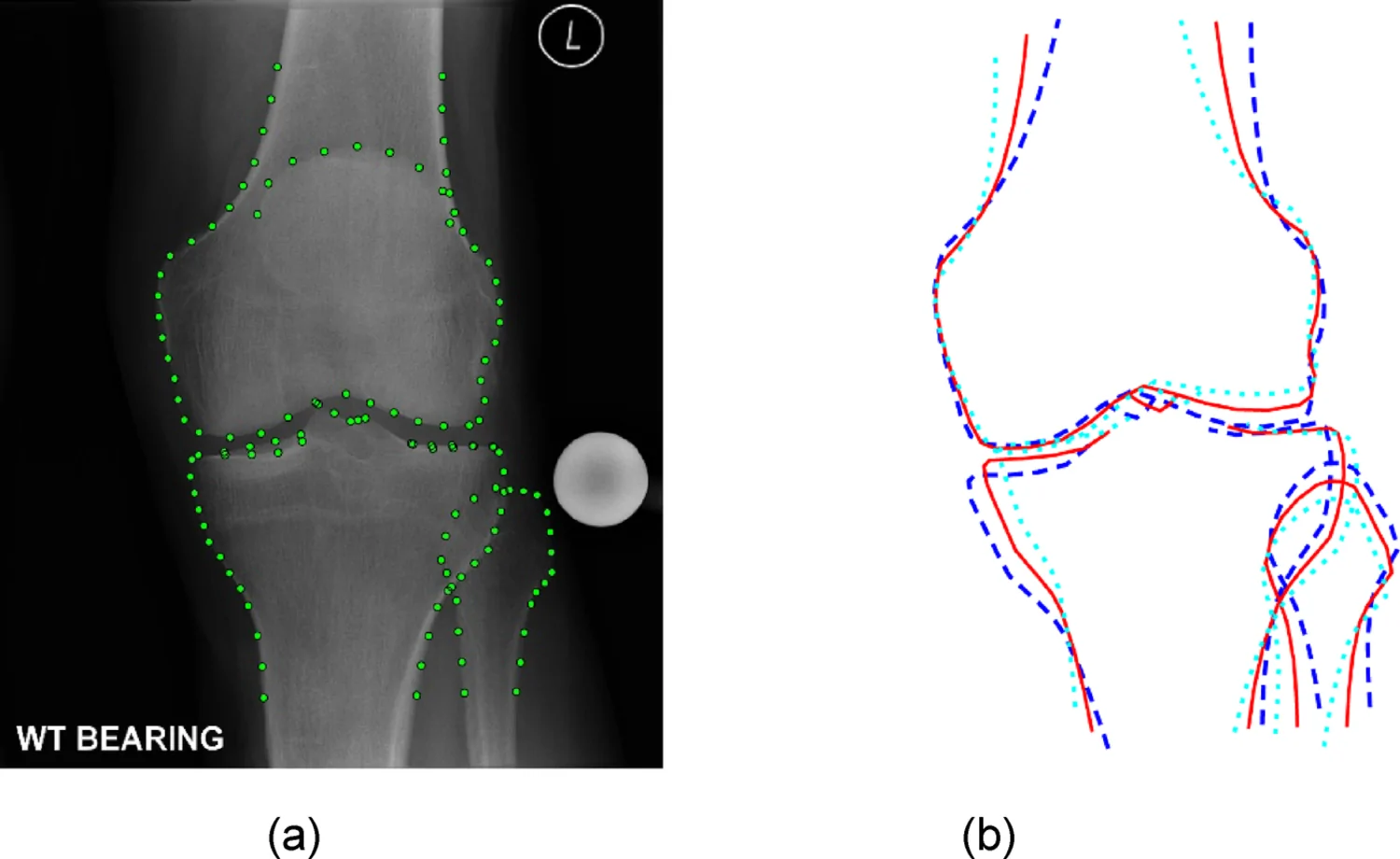
Statistical shape model (SSM) example: (**a**) Distal femur, proximal tibia and proximal fibula outlined with 110 points in an anteroposterior knee radiograph. (**b**) Visualisation of variation of first shape mode of SSM generated from 566 knee radiographs annotated with 110 points; red solid line shows mean shape with ± 2.5 standard deviations

SSMs capture the precise shape of objects using landmark points, which are human-defined points placed on key locations of the structure that are consistent across different instances of the same object. This could be, for example, the outline of the bones in the knee (see Fig. 5a). While each individual knee has its unique characteristics, the spatial relationships between the landmark points defining the bone shape will always lie within a constrained range. SSMs are primarily applicable to objects that follow a geometric template, where the relationships between landmark points follow a consistent pattern, such as bones or organs.

SSMs are created by taking a collection of images of the same structure (e.g. hips or knees), along with the associated landmark points for each image. These shapes are aligned into a common reference frame to remove differences in position, orientation and scale. The aligned point positions across all images are then processed to build an SSM by applying Principal Component Analysis (PCA) [29]. This results in a set of shape modes that capture the range of shape variation for the given structure (see Fig. 5b).

SSMs have been widely applied to study disease in a range of medical imaging applications [30]. In osteoporosis research, SSMs have been used to segment and reconstruct skeletal structures such as the femur and vertebrae, contributing to fracture risk prediction as well as vertebral fracture detection [31]. Recently, SSM-derived hip shape in UK Biobank was shown to be associated with incident hip fracture risk independently of established bone density and conventional geometric measurements [32].

Beyond their role in shape representation, SSMs can facilitate the automated detection of landmark points in new unseen images. This is typically achieved by integrating SSMs with search algorithms that use the encoded shape variation to constrain the search results, ensuring that the spatial configuration of all detected landmark points is plausible within the learned boundaries [33]. Early examples of shape model matching include Active Shape Models [34] and Active Appearance Models [35]. More recently, ML and deep learning approaches have been used to embed shape information for landmark detection [33, 36–39].

## Data Challenges for Clinical Application

For ML-based computer vision tools to perform reliably in clinical practice, they must be trained on data that is of a suitable quality and representative of the full scope of the likely data it will encounter in practice. In the context of osteoporosis, this includes imaging data from patients across a wide range of disease severity, anatomical variation, and demographic characteristics, as well as from healthy individuals. ML models trained using an insufficient subset of images (e.g. of a single sex, ethnicity or a limited age range) are prone to bias and may not function as required when applied to more diverse populations.

There are also technical challenges associated with the various imaging modalities used in clinical practice. It is generally the case that ML models trained on one modality (e.g. DXA, CT, MRI) will not function well (i.e. generalise) when applied to a different modality. Even within a single modality, performance can vary significantly depending on the scanner model, acquisitions parameters, and site-specific image acquisition protocols. These factors can introduce systematic biases, limiting the ML model’s ability to generalise to other data. Federated learning is a promising approach to mitigate this by using data from multiple sites for model training without the need for the data to be shared between sites [40, 41]. This does not only improve model accuracy through access to a wide pool of data but also preserves data privacy.

These challenges highlight the importance of ensuring that ML training data is both representative and technically consistent, in particular when developing and validating ML models intended for clinical application.

## The Importance of Validation

Once a ML model has been trained, thorough assessment is essential to demonstrate its performance across the range of data it is likely to encounter in clinical practice. During model development, a subset of data needs to be held-out to serve as a test dataset. The test dataset will be used to assess the performance of the model on data that was not used to develop and optimise the model. This will provide an unbiased evaluation.

In the context of ML, good generalisation is achieved when a trained ML model can successfully provide outputs from data that it has not seen before. If the model learns the characteristics of the training set too closely, it will perform well when evaluated on the training data but not on unseen test data, a circumstance known as overfitting. Avoiding overfitting helps to achieve good generalisation.

While internal validation using held-out test data provides an initial indication of model performance, it does not guarantee generalisability beyond the development dataset. Models may still be biased towards the characteristics of the training data, particularly if that data is limited in diversity or from a single site. To assess whether a model can generalise to diverse clinical populations and imaging conditions, independent external validation is required.

External validation is the process of assessing model performance on data created under similar but distinct conditions, for example, testing the model on data from a different hospital, or from a different scanner of the same modality. A model performing well on the original scanner but poorly on the latter may be overfitted to the original scanner’s characteristics. For ML tools to be used in clinical practice, external validation is essential to demonstrate robustness and to instigate trust into the performance of the system.

Both internal and external validation results should be carefully reviewed when selecting ML tools for clinical use, to ensure the assessment aligns with the target patient population and the imaging environment. Research shows that many clinical ML tools lack external validation, which limits the ability to assess their generalisability and raises concerns about their readiness for clinical deployment [42, 43]. In radiology, the Health AI Register (https://healthairegister.com/) has been established to improve the transparency around the validation of ML tools for clinical practice, including whether external validation has been performed and on what data. This important initiative not only serves as a decision aid for healthcare providers selecting clinical ML-based computer vision tools, but also raises awareness and emphasises the need for generating such validation data for any new tools.

## Conclusions and Future Directions

Computer vision methods are increasingly applied to medical imaging data, with the potential to support clinical decision-making, improve diagnostic consistency, and enable large-scale analysis. In the field of osteoporosis, evidence is emerging for the clinical utility of ML-based computer vision methods to predict fragility fractures [44], detect osteoporosis at the hip [45], and identify vertebral fractures [46]. Despite these advances, the clinical translation of these methods remains challenging. Key barriers include the limited availability of large, diverse datasets for training and testing, the lack of external validation for many existing tools, and difficulties with integrating ML-based computer vision tools into existing clinical workflows. Furthermore, issues related to interpretability, regulatory compliance, and clinician trust must be addressed to ensure safe and effective deployment.

Future work should focus on overcoming these limitations. For example, federated learning and privacy-preserving methods can help mitigate data-sharing constraints while maintaining patient confidentiality. In addition, explainable ML approaches will be essential for creating transparency and fostering clinician trust. The FUTURE-AI Consortium, a multi-stakeholder initiative, has outlined guiding principles for the development and deployment of trustworthy AI tools in healthcare, focussing on fairness, universality, traceability, usability, robustness, and explainability [47]. These principles are directly applicable to computer vision methods in osteoporosis, providing a framework to ensure that future tools are not only accurate but also transparent, equitable, and clinically usable.
